# Development of a one-step embryonic stem cell-based assay for the screening of sprouting angiogenesis

**DOI:** 10.1186/1472-6750-7-20

**Published:** 2007-04-16

**Authors:** Bastien Hermant, Agnès Desroches-Castan, Marie-Laure Dubessay, Marie-Hélène Prandini, Philippe Huber, Daniel Vittet

**Affiliations:** 1 Inserm, U882, Grenoble, F-38054 France; 2 CEA, DSV, iRTSV, Laboratoire Angiogenèse et Physiopathologie Vasculaire, Grenoble, F-38054 France; 3 Université Joseph Fourier, Grenoble, F-38054 France; 4 Inserm, U878, Grenoble, F-38054 France

## Abstract

**Background:**

Angiogenesis assays are important tools for the identification of regulatory molecules and the potential development of therapeutic strategies to modulate neovascularization. Although numerous in vitro angiogenesis models have been developed in the past, they exhibit limitations since they do not recapitulate the entire angiogenic process or correspond to multi-step procedures that are not easy to use. Convenient, reliable, easily quantifiable and physiologically relevant assays are still needed for pharmacological screenings of angiogenesis.

**Results:**

Here, we have optimized an angiogenesis model based on ES cell differentiation for screening experiments. We have established conditions leading to angiogenic sprouting of embryoid bodies during ES cell differentiation in type I three-dimensional collagen gels. Immunostaining experiments carried out during these cultures showed the formation of numerous buds comprising CD31 positive cells, after 11 days of culture of ES cells. Moreover, this one-step model has been validated in response to activators and inhibitors of angiogenesis. Sprouting was specifically stimulated in the presence of VEGF and FGF2. Alternatively, endothelial sprouting induced by angiogenic activators was inhibited by angiogenesis inhibitors such as angiostatin, TGFβ and PF4. Sprouting angiogenesis can be easily quantified by image analysis after immunostaining of endothelial cells with CD31 pan-endothelial marker.

**Conclusion:**

Taken together, these data clearly validate that this one-step ES differentiation model constitutes a simple and versatile angiogenesis system that should facilitate, in future investigations, the screening of both activators and inhibitors of angiogenesis.

## Background

Angiogenesis, the process of growth of blood capillaries from the pre-existing vascular tree, is a complex phenomenon that is either associated with or involved in the development of numerous physiological or pathological situations [1,2]. Among them, angiogenesis is considered crucial for revascularization after cardiac ischemia, and is also implicated in the pathogenesis of rheumatoid arthritis, diabetic retinopathy, and tumoral progression. In particular, numerous clinical and experimental data show that the growth of cancerous tumors and the formation of metastases are highly dependent on the establishment of tumoral neovascularization from the pre-existing vascular network [3]. The tumor microvascular network then represents a new target for cancer treatment and the identification and characterization of molecules that control the formation of blood vessels become of interest in the development of anti-cancer therapies. In addition, there is also a great interest in combining antiangiogenic therapy with other conventional cytotoxic therapies in cancer treatment since several studies have demonstrated that the delivery of therapeutics may be increased during vessel normalization induced by angiostatics [2].

Several angiogenesis regulators have now been identified and characterized [4]. Although first clinical trials of single agent antiangiogenic treatment have not always given satisfactory results, the use of an antiangiogenic therapy still remains highly promising in pathologies where angiogenesis is undesired [5]. In contrast, strategies aimed at stimulating angiogenesis could also present interest in many cases where neovascularization is needed such as after cardiac ischemia or after tissue graft. Then, there is a great challenge to find new potential angiogenesis activators or inhibitors that may be candidate for therapeutics. Within this context, the setting up of models for screening active molecules (angioactive or angiostatic) on the angiogenic response, is of considerable importance. Numerous in vitro angiogenesis models have been developed [6,7]. They are either two-dimensional (2D) models such as conventional cell proliferation and migration tests or more elaborated three-dimensional (3D) assays. Concerning 3D angiogenesis models, assays involving Matrigel, a matrix derived from mouse Engelbreth-Holm-Swarm sarcoma, are among the most common commercially available in vitro angiogenesis assays. Other 3D models are based on the use of fibrin or type-1 collagen as a support matrix for endothelial cells. However, both 2D and 3D models mostly involve the study of one particular step of the angiogenic response, but do not recapitulate the entire angiogenic process including proliferation, migration and tubulogenesis. Although they exhibit some interest for primary screening because of their simplicity, an assay recapitulating all the sprouting angiogenic process should be preferable since it would be more physiologically relevant. Other models that more closely recapitulate the sprouting angiogenic response have therefore been established. They include models based on the 3D culture of endothelial cell-coated microcarriers or endothelial cell spheroids embedded in collagen gels [8,9]. However, they require multi-step procedures and are not easy to perform. Mouse embryonic stem cells (ES cells) have also been shown to be a good alternative as a system for the study of differentiation towards the endothelial lineage [10-14]. In addition, this cellular system, into which genetic modifications can easily be introduced, can go through most of the stages of budding angiogenesis as observed in vivo [15-17]. In the previously described ES cell model, two steps are required for angiogenesis to proceed [15]. First, ES cells are induced to differentiate into embryoid bodies (EBs). EBs are then collected and further cultured into a type I 3D collagen matrix for another period, during which the EB primary vascular structures extend and invade the collagen matrix, leading to complex and ramified endothelial sprouts mimicking the sprouting angiogenesis process. This two-step model makes possible to carry out qualitative and quantitative studies by cell imaging [15]. All of these characteristics together, therefore, make it a powerful study model that enables to evaluate, *in vitro*, the efficiency of active molecules with respect to both vascular development and angiogenesis, and to improve the understanding of the molecular mechanisms that are called into play during these responses.

Ideally, an in vitro angiogenesis assay suitable for the screening of potentially active compounds should be convenient, reliable, easily quantifiable and physiologically relevant. In addition, it should allow evaluation of multiple samples in one experiment. Although the ES cell-based angiogenesis model fills most of these requirements, it remains time consuming since it proceeds in two culture steps and requires many manual interventions before being processed for angiogenesis visualization and quantification that may constitute an inconvenient for screening experiments.

Matrix proteins, including type I collagen, support cell differentiation in many lineages [18]. A recent study indicated that ES cells can undergo multi-lineage differentiation including differentiation into cells of the endothelial lineage when plated directly into collagen gels [19]. A 3D collagen environment for ES cell differentiation then appears to constitute a mean to improve ES two-step angiogenesis models. Moreover, some early unpublished data have also postulated that ES cell differentiation into a mixture of type I and type III collagens could be used for the screening of angiogenesis regulating factors [20]. In this context, the aim of the present work was to simplify the *in vitro *differentiation model of ES cells in order to establish and to validate a one-step model that may be relevant for the preclinical screening of active molecules on angiogenesis.

## Results

**Figure 1:**
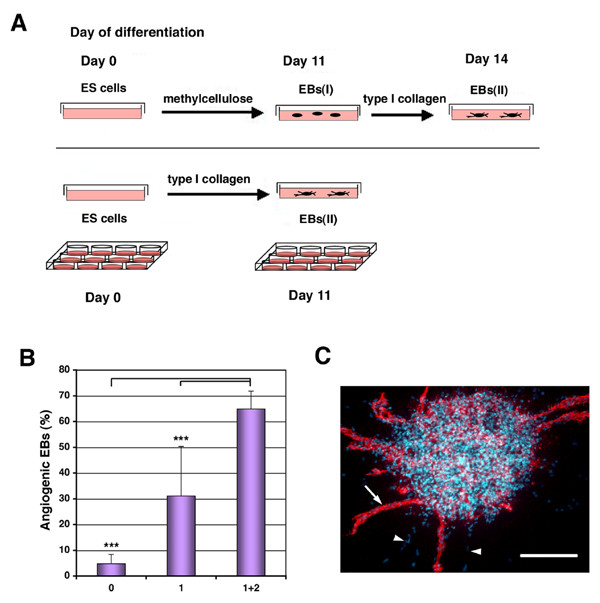
**Development of angiogenic embryoid bodies during one-step ES cell differentiation into type I collagen gels**. A, Schematic illustration of the one-step method, performed either in 35 mm-diameter plates or in 12-well microplates, compared with the two-step initial angiogenesis model; B, Effect of the addition of angiogenic growth factors (VEGF + FGF2) on the formation of EBs exhibiting endothelial sprouts (angiogenic EBs). ES cell differentiation was performed in the absence (0) or in the presence (1) of angiogenic growth factors added at the initiation of the differentiation of CJ7 ES cells at day 0. (1+2), effect of a second angiogenic growth factor addition at day 6, in addition to day 0, on the formation of sprouting angiogenic EBs. *** p < 0.001; mean values significantly different by unpaired Student's *t *test. C, CD31 immunostaining of a 11 day-old angiogenic embryoid body derived from CJ7 ES cells obtained in the one-step differentiation method. The arrow points to a CD31-positive endothelial sprout (red staining) and arrowheads to CD31-negative sprouts that only exhibit blue staining of cell nuclei after Hoescht 33258 counterstaining. Scale bar = 200 μm.

In order to simplify the two step ES-based angiogenesis initial model, a first series of experiments was designed to test the ability of type I collagen to support the formation of EBs and of endothelial sprouts. A type I collagen gel was used since previous works have shown that sprouting angiogenesis can develop from EBs embedded in this matrix [15,16,21], and since type I collagen was observed to support multilineage ES cell differentiation [19]. ES cells were directly seeded into type I collagen gels in the presence of angiogenic factors (50 ng/ml VEGF and 100 ng/ml FGF2) in 35 mm-diameter dishes or in 12-well microplate format (Figure 1A). VEGF was added since it has been proven efficient for both ES cell endothelial differentiation [12,22,23] and ES-derived EB sprouting angiogenesis [15]. In addition to the regulation of the early stages of mesoderm induction and angioblast formation [24], FGF2 was also shown to increase vascular development in the embryoid body model [12,25,26]. In these conditions, formation of EBs was observed within a few days, as previously described in methylcellulose semi-solid differentiation medium [15,16]. First signs of EB sprouting were observed after day 6 of differentiation. These sprouts further extended and developed a complex cellular network after 11 days of differentiation. CD31 immunostaining experiments revealed that many sprouts were constituted of endothelial cells. EBs exhibiting CD31-positive sprouts were termed angiogenic. In the presence of both VEGF and FGF2, added at the initiation of the culture, near 30% of EBs (31.1 ± 19.3%, SD, n = 5) displayed an angiogenic phenotype, whereas less than 5% of EBs (4.8 ± 3.6%, SD, n = 2) were found angiogenic when no exogenous growth factor addition was done (Figure 1B). To improve the number of angiogenic EBs, repeated addition of angiogenic factors were tested during the course of ES cell differentiation in collagen gels. We have found that a second growth factor addition at day 6 of differentiation greatly improved the development of angiogenic EBs to a level similar to that observed in the initial two-step model [15]. In this condition, 80 to 100 EBs (88 ± 28, SD, n = 19) can form from 2,000 ES cells when initially plated in 35 mm-diameter dishes, and we observed that more than 60% of EBs (64.9 ± 7%, SD, n = 25) displayed a sprouting angiogenic phenotype when two growth factor additions were performed (Figure 1B). A representative sprouting angiogenic EB is illustrated in Figure 1C.

**Figure 2:**
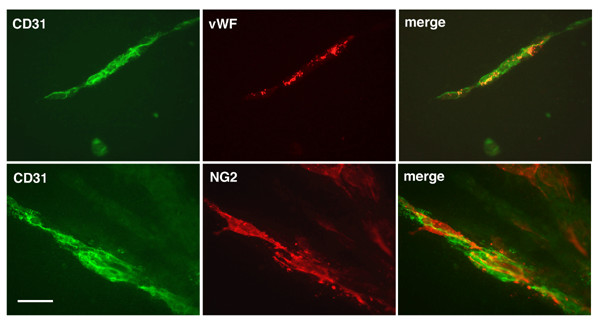
**Double immunostainings of embryoid bodies sprouts for CD31, von Willebrand factor (vWF) and NG2 proteoglycan**. CJ7 ES cells were allowed to differentiate in the presence of angiogenic growth factors (VEGF+FGF2) added at day 0. EBs angiogenic sprouts were analyzed at day 11 of differentiation. vWF immunoreactivity (red fluorescence) located in Weibel-Palade bodies can be observed in several sprouting CD31-positive cells (green fluorescence) (upper panels). Elongated NG2 proteoglycan-positive cells (red fluorescence) can be seen close to CD31-positive cells constituting endothelial sprouts (lower panels). Scale bar = 50 μm.

In order to characterize the maturation level that can be achieved in endothelial sprouts, we then looked at vWF expression, a marker characteristic of mature endothelial cells, and investigated whether pericytes may be associated with endothelial sprouts by looking at the expression of NG2 proteoglycan. NG2 proteoglycan has been shown to be expressed by nascent pericytes during development [27]. Such an association between endothelial cells and vascular mural cells was previously described in two steps more complex ES cell-based differentiation models [22,15,21]. We observed that several cells located in the CD31-positive sprouts contained vWF, as shown by co-immunofluorescence studies (Figure 2, upper panels). Analysis of EB sprouts also revealed that several NG2 proteoglycan-positive cells can be found in the vicinity of the endothelial cells constituting the sprouts (Figure 2, lower panels).

**Figure 3:**
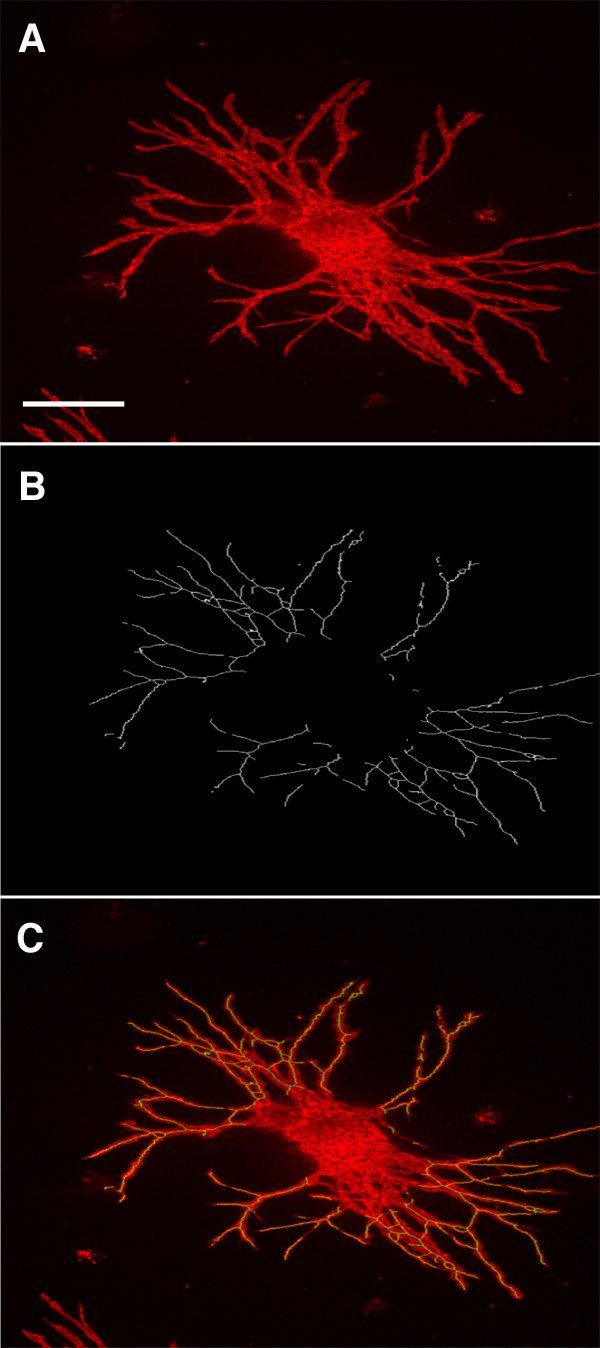
**Image processing for quantitation of endothelial sprouting using MetaMorph Offline software**. A, Initial image of the CD31 staining of an 11-day-old angiogenic EB derived from CJ7 ES cells; B, Skeletonized image of image A; C, Superposition of image B and image A. Scale bar = 400 μm.

**Figure 4:**
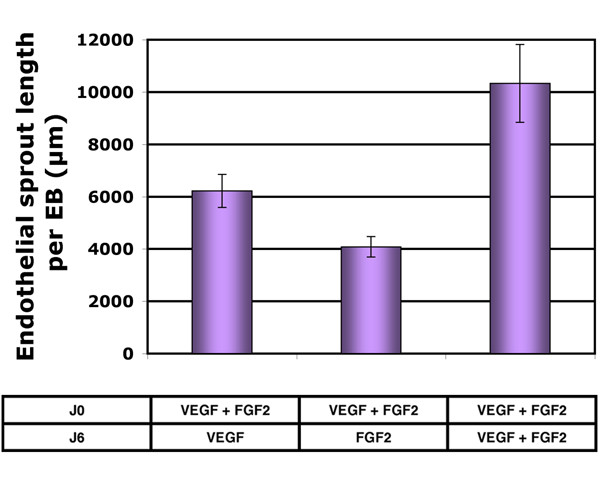
**Quantitative analysis of endothelial sprouting in response to FGF2 and VEGF**. CJ7 ES cells were first induced to differentiate at day 0 in the presence of both VEGF (50 ng/ml) and FGF2 (100 ng/ml). For the second growth factor addition at day 6, VEGF (50 ng/ml) and FGF2 (100 ng/ml) were added alone or in combination. The mean total sprout length of EBs exhibiting endothelial sprouts (angiogenic EBs) was measured at day 11, after CD31 immunostaining, and image analysis with MetaMorph Offline software. Data represent the mean values ± SD resulting from at least 25 angiogenic EBs obtained in one differentiation experiment.

To validate the one-step model as a screening assay for both activators and inhibitors of angiogenesis, we examined whether known angiogenesis regulators were able to affect the sprouting response. After CD31 immunostainings, EBs exhibiting endothelial CD31-positive sprouts were selected for image capture. In each experiment, at least 15 different EBs and in most cases more than 24 EBs were analyzed per condition of test. The extent of the EB endothelial sprouting was quantified by morphometric analysis using the MetaMorph software. Figure 3 illustrates the result of the image processing with the MetaMorph software. We first analyzed the respective contribution of VEGF and of FGF2 on the EB endothelial sprouting response. For this experimental series, ES cell differentiation was initiated in the presence of both VEGF and FGF2 added at day 0. The EB endothelial sprouting responses induced by the addition at day 6 of optimal VEGF and FGF2 concentrations [15], used alone or in combination, were quantified at day 11. We found that VEGF plays a key role in the formation of endothelial sprouts since its addition was responsible for approximately 60% of the EB endothelial sprouting response obtained when both VEGF and FGF2 were present (Figure 4). The contribution of FGF2 was lower than that of VEGF, and the effects of the two factors were found to be additive (Figure 4).

**Figure 5:**
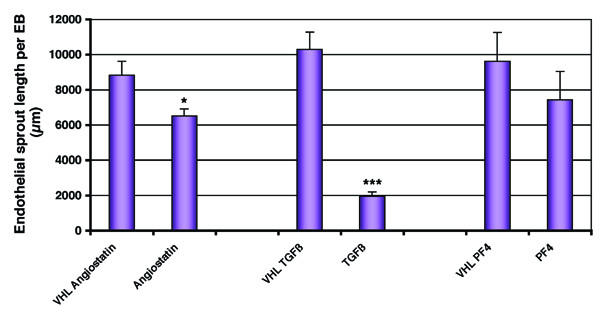
**Quantitative analysis of endothelial sprouting in response to angiogenesis inhibitors**. For inhibition studies of EB endothelial sprouting, VEGF (50 ng/ml) and FGF2 (100 ng/ml) were added at day 0 and at day 6 as indicated in the Methods. At day 6, EBs were also treated by the indicated angiogenesis inhibitors or by their respective vehicle (VHL). Analysis of the mean total sprout length of angiogenic EBs was performed at day 11 on 39, 25, 26, 18, 55 and 76 EBs treated with VHL TGFβ, 10 ng/ml TGFβ, VHL PF4, 2.5 μg/ml PF4, VHL angiostatin and 2.5 μg/ml angiostatin, respectively. In each condition, data represent the mean values ± SE from one out of two differentiation experiments performed in collagen gel with CJ7 ES cells. * p < 0.05, *** p < 0.001; significantly different from respective control values by unpaired Student's *t *test.

We also evaluated whether agents exhibiting known antiangiogenic activity were effective in this system. Several angiogenesis inhibitors are now identified and characterized including matrix-derived and non-matrix-derived compounds [28]. Among these two categories, Platelet Factor 4 (PF4), angiostatin (kringle1–4) and Transforming Growth Factor β1 (TGFβ) were tested on the endothelial sprouting response induced by angiogenic factors (50 ng/ml VEGF and 100 ng/ml FGF2). Quantitative analysis revealed that 2.5 μg/ml PF4 decreased the length of endothelial sprouts. Moreover, treatment with 2.5 μg/ml angiostatin or with 10 ng/ml TGFβ resulted in highly significant inhibition of the mean length of total endothelial sprouts per EB (Figure 5).

## Discussion

In this paper, we report the development and the validation of a simple one-step ES cell differentiation model for the screening of angiogenesis regulators. We provide evidence that this one-step model can recapitulate the various stages of sprouting angiogenesis, as initially reported for previously established two-step ES differentiation models. Moreover, we describe a standardized procedure to obtain reproducible and efficient development of endothelial sprouts from EBs using this one-step model. Compared with the two-step procedure, only one growth factor addition is required after initial seeding without any further intervention, which greatly simplifies the test. In comparison with other available in vitro models, the one-step ES cell based angiogenesis model shows the major advantage of reproducing at least three aspects of the sprouting angiogenesis response: proliferation, migration and vascular morphogenesis. As previously observed in two-step ES cell differentiation procedures [22,15,21], the vascular endothelial sprouts that develop in the one-step ES cell model are associated with pericytes, which confers to the one-step model superiority to many in vitro angiogenesis models that mostly recapitulate only limited aspects of the angiogenic response [7].

Multiple steps of the angiogenic process may be altered by an angiogenesis-targeted drug. The fact that the ES differentiation model mimicks the complexity of the sprouting angiogenesis response makes it relevant for further identification of novel molecules and mechanisms regulating this process. EB endothelial sprouting was specifically induced by angioactive molecules, namely VEGF and to a minor extent FGF2, and inhibited by known angiostatic factors. These data validate the one-step ES differentiation model as a versatile angiogenesis system for the screening of both activators and inhibitors of angiogenesis.

The ES differentiation one-step procedure is easier to use than the other 3D angiogenesis existing models since they all proceed into two steps and require extensive manipulations. Screening experiments can be performed in both 6-well or 12-well-microplaque format that could be very useful for further adaptation of the model for automatization and may be convenient to further characterize molecules identified by primary screens using less complete cellular models.

## Conclusion

Thus, the one-step ES-based angiogenesis model, that allows routine and reproducible accomplishment in a reduced time, constitutes a good alternative for existing in vitro angiogenesis models. In addition, in view of its simplicity, it allows an easy transfer to non-specialist investigators. We expect the one-step ES angiogenesis model to be particularly valuable to extend drug discovery screening.

## Methods

### Embryonic Stem (ES) cell differentiation

CJ7-ES cells [29], and on rare occasions R1-ES cells [30], were used. ES cells were cultured on gelatin-coated cell culture dishes and were maintained undifferentiated in the presence of 10^3 ^U/ml Leukemia Inhibitory Factor (LIF) (ESGRO, Chemicon, Temecula, CA) as previously described [12]. At day 0, to initiate the differentiation, cells were collected by trypsin, counted and resuspended in Iscove modified Dulbecco's medium (IMDM), before being gently mixed into a type I collagen-medium in the presence of angiogenic factors: 50 ng/ml VEGF (Peprotech, Rocky Hill, NJ) and 100 ng/ml FGF2(a generous gift from Dr Ana Maria Gonzales, Queen Elizabeth medical center, Birmingham, UK). Final composition of the collagen-medium was 15% fetal calf serum (FCS) (Biowest, Nuaillé, France), 450 μM of monothioglycerol, 1.2 mg/ml type I collagen (BD Biosciences, Bedford, MA) neutralized with 1 N NaOH (according to the manufacturer recommendation), 10 μg/ml insulin and 0.5% penicillin-streptomycin. Practically, final ES cell suspensions in the collagen medium were obtained by mixing ES cells in collagen solution with a concentrated solution of medium as previously detailed in the procedure for EB secondary culture into type I collagen gels [31]. ES cells in collagen-medium were poured either in 35-mm bacterial dishes (1.5 ml per well) or in 12-well bacterial microplaque (1 ml per well) at a concentration of 2,000 cells/well, leading to the obtention of a 1.5 mm-thick or a 2.4 mm-thick gel, respectively. The cells were incubated at 37°C, in a 5% CO_2_, 95% air atmosphere. At day 6, a second addition of VEGF and FGF2 was done using the same concentrations of growth factors as used at day 0. Factors were diluted either into 100 μl or into 200 μl medium lacking collagen before being added to 12-well plates or 35-mm dishes, respectively. Cultures were stopped at day 11 for EBs analysis.

### Immunofluorescence

At day 11 of differentiation, collagen gels containing EBs were collected on glass slides. Dehydration was obtained using nylon linen and adsorbent cards, thus allowing 2D projection of the sprouts. EBs were then fixed using 3% paraformaldehyde in PBS for 15 minutes at room temperature. After three washes with PBS, slides were incubated 1 hour at room temperature with rat anti-mouse CD31 monoclonal antibody MEC-13.3 [32], (a generous gift from Dr A. Vecchi, Mario Negri Institute, Milan, Italy), used as undiluted hybridoma supernatant. After 3 rinses, slides were incubated for one hour at room temperature with cyanine 3-conjugated goat anti-rat IgG (Jackson Immunoresearch Laboratories, West Grove, PA) diluted 1:300. After three further washes in PBS, slides were counterstained with Hoescht 33258. Slides were mounted in Fluorsave (Calbiochem, La Jolla, CA).

In some experiments, EBs were double labeled with rat anti-mouse CD31 monoclonal antibody MEC13.3, used as undiluted hybridoma supernatant, and rabbit antibodies directed against rat proteoglycan NG2 (1 μg/ml) (a generous gift from Dr W.B. Stallcup, La Jolla, CA) or against human von Willebrand factor (vWF) (DakoCytomation, Trappes, France) (dilution 1:500), and the staining was revealed by incubation with a mixture of cyanine 3-conjugated goat anti-rabbit IgG (Jackson Immunoresearch Laboratories) and of Alexa488-conjugated goat anti-rat IgG (Molecular Probes, Eugene, OR). When immunofluorescence experiments were performed with vWF antibodies, EBs were fixed and permeabilized with 3% paraformaldehyde and 0.5% Triton X-100 for 3 min, followed by a supplementary 15 min fixation with 3% paraformaldehyde alone, before rinses with PBS and immunostainings.

### Angiogenesis Inhibitors

When tested, angiogenesis inhibitors were added at day 6 at the same time of the second angiogenesis activators addition. They were used at optimal concentrations known to affect endothelial sprouting from EBs in the two-step ES cell angiogenesis model [15,33]. Platelet factor 4 (PF4) (Diagnostica Stago, Asnières, france) was used at 2.5 μg/ml; angiostatin (fragment K1–4) (Calbiochem) was used at 2.5 μg/ml and Transforming Growth factor β1 (TGFβ) (R&D Systems, Abingdon, UK) was used at 10 ng/ml. Inhibitors were added with angiogenesis growth factor activators (VEGF and FGF2) diluted in differentiation medium to each well (100 μl) or dishes (200 μl).

### Image analysis

After dehydration of the collagen gels to allow the complete 2D projection of the sprouts and immunostaining with anti-CD31 antibodies, the slides were observed using a Zeiss Axioplan fluorescence microscope (Zeiss, Oberkochen, Germany). Images were captured, at 5× or at 10× magnification, depending on the size of the sprouting EBs, with a computer-supported digital Spot RT camera using Spot advanced software (Diagnostic instruments, Sterling Heights, MI). In order to quantify the sprouting, images were processed for morphometric analysis using Metamorph Offline software (Molecular Devices, Downingtown, PA). A macrocommand was edited to give the total endothelial sprout length of each EB after binarization and skeletonization of the image of the CD31 staining.

## Authors' contributions

HB developed strategies and carried out experiments to improve the detection of endothelial sprouts. He also participated in all other aspects of the experimental work.

AD-C performed the experiments to establish the one step procedure. She developed the quantification procedure and carried out the quantitative analysis with angiogenesis inhibitors for the validation of the assay. Both HB and AD-C participated in the analysis of the data and collaborated in the drafting of the manuscript.

MLD participated in the establishment of the culture conditions for the formation of angiogenic embryoid bodies during the one step culture.

MHP contributed in the design of molecular biology experiments and was involved in experimental support.

PH is the head of the laboratory. He contributed intellectually at the initiation of the study and has been involved in revising the manuscript.

DV coordinated the activity of the research group. He performed the experiments at the beginning of the study and drafted the manuscript.

All authors read and approved the final manuscript.
